# The NMR signature of gluconoylation: a frequent N-terminal modification of isotope-labeled proteins

**DOI:** 10.1007/s10858-019-00228-6

**Published:** 2019-02-08

**Authors:** David Schweida, Pierre Barraud, Christof Regl, Fionna E. Loughlin, Christian G. Huber, Chiara Cabrele, Mario Schubert

**Affiliations:** 1 Department of Biosciences, University of Salzburg, Billrothstr. 11, 5020 Salzburg, Austria; 2 Institute of Molecular Biology and Biophysics, ETH Zürich, 8093 Zurich, Switzerland; 3 Institut de Biologie Physico-Chimique (IBPC), UMR 8261 CNRS, Université Paris Diderot, 13 rue Pierre et Marie Curie, 75005 Paris, France; 4 Christian Doppler Laboratory for Innovative Tools for Biosimilar Characterization, University of Salzburg, Hellbrunnerstrasse 34, 5020 Salzburg, Austria; 5 Department of Biochemistry & Molecular Biology, Monash Biomedicine Discovery Institute, Monash University, Clayton, VIC 3800 Australia

**Keywords:** NMR spectroscopy, Post-translational protein modification, Gluconoylation, Gluconic acid, N-terminus, Recombinant protein, *E. coli*

## Abstract

N-terminal gluconoylation is a moderately widespread modification in recombinant proteins expressed in *Escherichia coli*, in particular in proteins bearing an N-terminal histidine-tag. This post-translational modification has been investigated mainly by mass spectrometry. Although its NMR signals must have been observed earlier in spectra of ^13^C/^15^N labeled proteins, their chemical shifts were not yet reported. Here we present the complete ^1^H and ^13^C chemical shift assignment of the N-terminal gluconoyl post-translational modification, based on a selection of His-tagged protein constructs (CCL2, hnRNP A1 and Lin28) starting with Met-Gly-...-(His)_6_. In addition, we show that the modification can hydrolyze over time, resulting in a free N-terminus and gluconate. This leads to the disappearance of the gluconoyl signals and the appearance of gluconate signals during the NMR measurements. The chemical shifts presented here can now be used as a reference for the identification of gluconoylation in recombinant proteins, in particular when isotopically labeled.

**Electronic supplementary material:**

The online version of this article (10.1007/s10858-019-00228-6) contains supplementary material, which is available to authorized users.

## Introduction

**Fig. 1:**
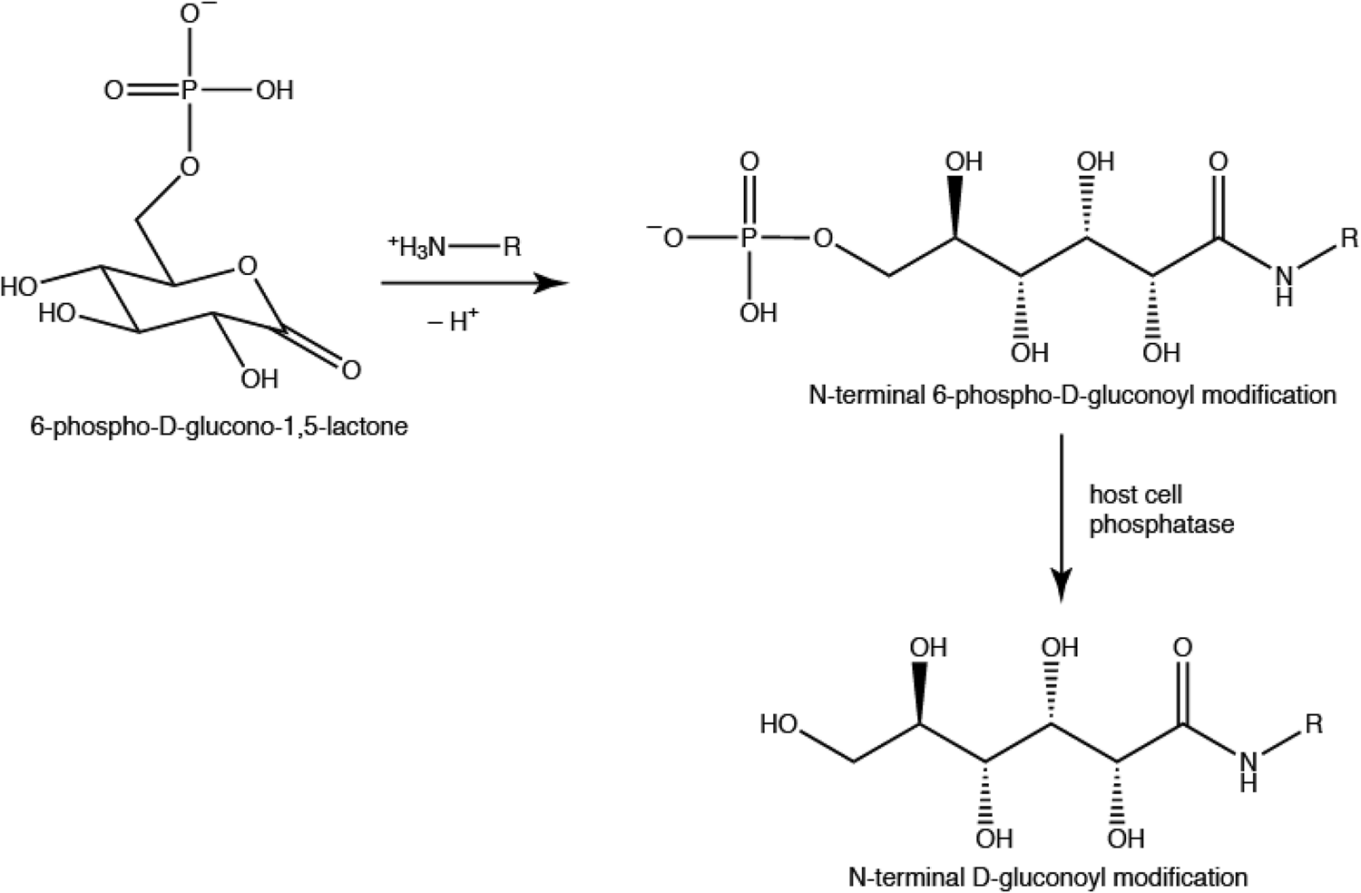
Mechanism of gluconoylation according to Geoghegan et al. (1999), in which the metabolite 6-phospho-glucono-1,5-lactone, originating from glucose-6-phosphate, reacts spontaneously with a free N-terminus of a protein

Post-translational protein modifications (PTMs) add another layer of complexity to the proteome and each modification can potentially change the structure, function and stability of a protein (Aebersold et al. 2018). For recombinant proteins, knowledge about the protein homogeneity and the presence of PTMs is crucial for most applications, e.g. pharmaceutical applications. N-terminal gluconoylation and phosphogluconoylation were first noticed by mass spectrometry in recombinant proteins expressed in *Escherichia coli* (Geoghegan et al. 1999; Yan et al. 1999a). The phosphogluconoyl moiety is formed by a spontaneous reaction of the metabolite 6-phosphogluconolactone with free N-termini of proteins (Fig. 1). The phosphate group is typically cleaved by host cell phosphatases leading to gluconoyl. The occurrence of these modifications depends on the N-terminal sequence, and likely on the bacterial strain and the bacterial growth conditions. BL21(DE3), the most commonly used *E. coli* strain for protein expression, is known to accumulate 6-phosphogluconolactone due to the lack of 6-phosphogluconolactonase (Meier et al. 2012), which favors gluconoylation, so that it is not unexpected that this strain produces significant amounts of gluconoylated proteins. It was shown previously that gluconoylation occurs with many N-terminal histidine-tagged proteins (Geoghegan et al. 1999; Yan et al. 1999a; Du et al. 2005; She et al. 2010; Martos-Maldonado et al. 2018) with N-terminal sequences that are also found in widely used, commercially available expression vectors. However, one protein that did not contain an N-terminal histidine-tag was also reported to be highly susceptible to gluconoylation (Aon et al. 2008). The *E. coli* methionine aminopeptidase (MAP) is an essential enzyme involved in protein N-terminal methionine excision. This enzyme is very well known for cleaving all proteins with small side chains on the residue directly following the N-terminal methionine (Flinta et al. 1986). For instance, proteins with Ala, Gly or Ser at the second amino-acid position are very efficiently processed by MAP (Frottin et al. 2006), and the gluconoyl group is thus attached to the second residue in that case (Yan et al. 1999b). Recombinant proteins expressed in M9 minimal medium seems to yield higher amounts of gluconoyl (Yan et al. 1999a) compared to Luria broth medium (Geoghegan et al. 1999; She et al. 2010), which is of special interest for the protein NMR community, because M9 minimal medium is routinely used for isotope labeling. Gluconoylation is highly selective for N-termini, as shown by the treatment of model peptides and enhanced green fluorescent protein (EGFP) with gluconic acid δ-lactone that led only to gluconoylation at the N-terminus but not at the ε-amino group of Lys side chains (Martos-Maldonado et al. 2018).

Here we present the NMR chemical shifts of gluconoyl, which result in a characteristic signature in ^1^H–^13^C-HSQC spectra, as illustrated by the spectra of *Coprinopsis cinerea* lectin 2 (CCL2) (Schubert et al. 2012), two domains of the RNA-binding protein hnRNP A1 (Barraud and Allain 2013) and the tandem zinc knuckles of pluripotency factor Lin28 (Loughlin et al. 2012). In addition, we observed that gluconoyl is cleaved over time at conditions like pH 5.8 and 310 K, which leads to the formation of gluconate and a free N-terminus in longer NMR experiments. With the here presented chemical shift assignments, both N-terminal gluconoyl and gluconate can be readily identified in NMR spectra.

## Materials and methods

### Protein expression

The lectin CCL2 was expressed using a pET22b vector as described earlier (Schubert et al. 2012). Either Luria broth (Thermo Fisher Scientific) or M9 minimal medium (Sambrook 2001) with or without ^13^C and ^15^N isotope-labeling was used as culture medium. After affinity chromatography purification the buffer was exchanged to 50 mM KH_2_PO_4_/K_2_HPO_4_ pH 5.8, 150 mM NaCl by dialysis (3.5 kDa cutoff, Spectra/Por) and the proteins were concentrated with ultrafiltration devices (3 kDa cutoff, Amicon/Millipore or Vivaspin/Satorius). Most CCL2 spectra were recorded without ligand, but few were in complex with the trisaccharide GlcNAcβ1,4[Fucα1,3]GlcNAcβO(CH_2_)_5_COONa at pH 4.7. The individual domains of the RNA-binding protein hnRNP A1 were expressed and purified as described previously (Barraud and Allain 2013). Both domains were individually studied in complex with RNA, the RNA-recognition motif 1 (RRM1) in complex with the RNA UUAGGUC and RRM2 with the RNA UCAGUU in 10 mM NaH_2_PO_4_/Na_2_HPO_4_ pH 6.5 as described earlier (Beusch et al. 2017). The tandem zinc-knuckles of Lin28 (amino acids 124–186) were expressed, purified and complexed with AGGAGAU RNA from pre-miRNA let-7 as described (Loughlin et al. 2012). Spectra of the Lin28-RNA complex were measured in 10 mM sodium acetate pH 5.6, 1.5 mM β-mercaptoethanol and 0.15 mM ZnCl_2_ at 303 K.

### NMR spectroscopy

All spectra were recorded on Bruker Avance III spectrometers operating at 500, 600, 750 or 900 MHz, equipped with TCI, TXI or QXI probes at either 310 K or 303 K. Standard 2D spectra like ^1^H–^13^C HSQC, ^1^H–^15^N HSQC were routinely measured. A 2D constant time ^1^H–^13^C HSQC was recorded with 26.6 ms (Vuister and Bax 1992). A 3D HC(C)H-COSY (Gehring and Ekiel 1998) was recorded with 512 × 37 × 158 complex points, t_1max_ = 18.9 ms, t_2max_ = 2.79 ms, eight transients. A 3D (H)CCH-TOCSY (Bax et al. 1990) was recorded with 512 × 64 × 54 complex points, t_1max_ = 5.1 ms, t_2max_ = 6.1 ms, 16 transients and a mixing time of 23 ms. Spectra were referenced to 2,2-dimethyl-2-silapentanesulfonic acid (DSS) using an external sample of 0.5% DSS and 2 mM sucrose in H_2_O/D_2_O (Bruker), and indirect chemical shift referencing for ^13^C and ^15^N according to IUPAB (Markley et al. 1998). Data was processed with Topspin 3.2 (Bruker, Germany) and analyzed with Sparky (T.D. Goddard and D.G. Kneller, SPARKY 3, University of California, San Francisco, USA).

### Mass spectrometry

For analysis by mass spectrometry CCL2 samples without ^13^C and ^15^N isotope-labeling were diluted to 1.0 µmol L^−1^ in H_2_O + 0.10% formic acid (FA, Sigma-Aldrich GmbH, Steinheim, Germany). Ultrapure water was produced in house by a Milli-Q System (Millipore Corporation, Billerica, USA). Chromatographic separation of intact CCL2 proteins was carried out on an UltiMate 3000 Rapid Separation System (U3000 RSLC, Thermo Fisher Scientific, Germering, Germany) at a flow rate of 200 µL min^−1^ using a ProSwift RP-10R column (500 × 1.0 mm i.d., Thermo Fisher Scientific, Sunnyvale, CA, USA), operated at a temperature of 70 °C. Mobile phase A was H_2_O + 0.10% FA, mobile phase B was composed of acetonitrile (VWR, Leuven, Belgium) + 0.10% FA. The gradient applied was: 20% B for 2 min, 20–50% B in 2 min, 80% B for 2 min and 20% B for 5 min. Injection was carried out in in-line split-loop mode, injection volume was 10 µL (1.0 pmol). UV-detection was carried out at 214 nm with a 2.5 µL flow cell. Mass spectrometry was performed on a benchtop quadrupole-Orbitrap instrument (Q Exactive™) equipped with an Ion Max™ source with a heated electrospray ionization (HESI) probe, both from Thermo Fisher Scientific (Bremen, Germany). The instrument settings were as follows: spray voltage of 4.0 kV, sheath gas flow of 15 arbitrary units, auxiliary gas flow of 5 arbitrary units, capillary temperature of 275 °C, S-lens RF level of 60.0, AGC target of 5e6 and a maximum injection time of 200 ms. The samples were analyzed with full scans at a scan range of *m*/*z* 800–2600 and a resolution setting of 140,000 at *m*/*z* 200. The Xcalibur 3.0 software with the integrated Xtract algorithm was employed for deconvolution of raw mass spectra into zero charge-state spectra.

## Results

### Chemical shift correlations of the gluconoyl group

**Fig. 2:**
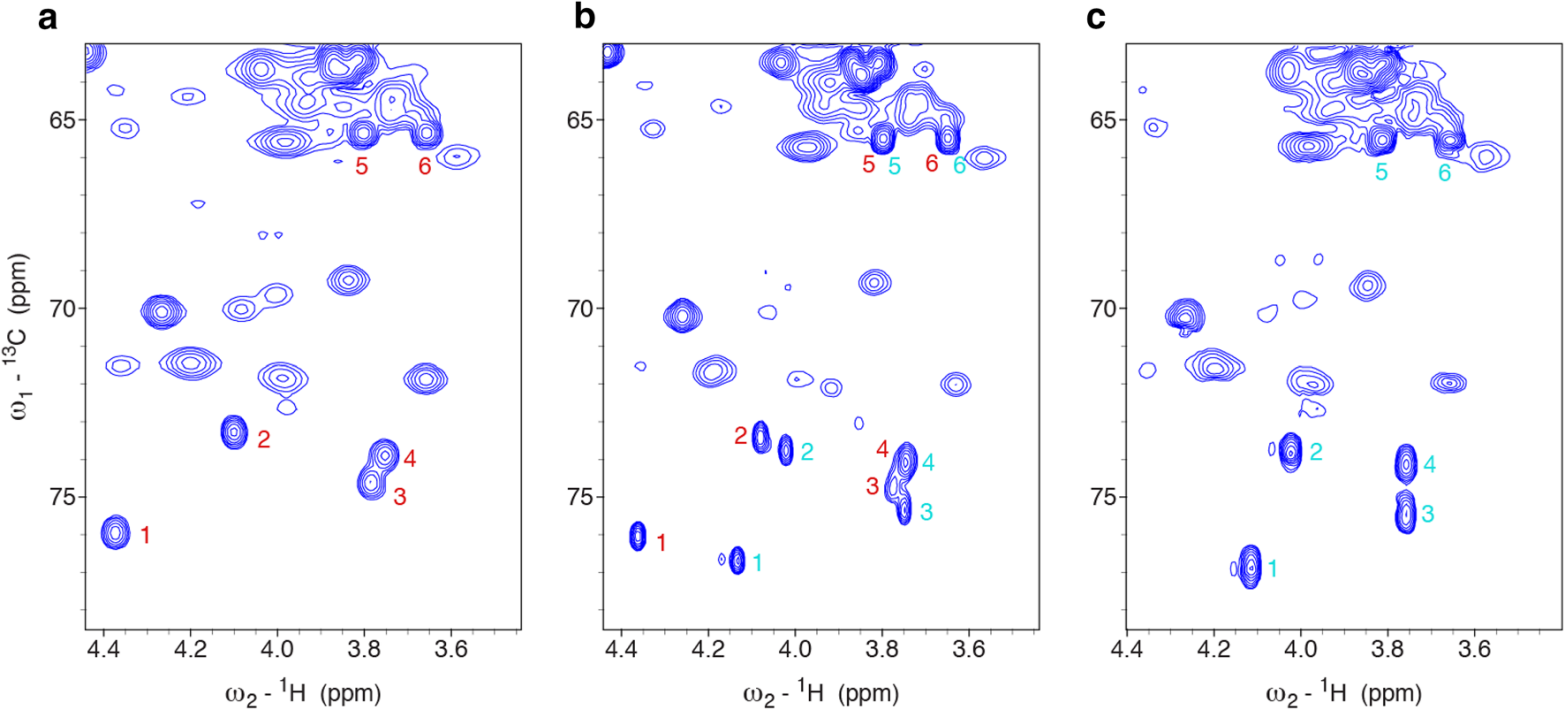
Chemical shift correlations of the PTM observed in the lectin CCL2, illustrated with ^1^H–^13^C HSQC spectra, change with time. **a** Spectrum of a fresh ^13^C/^15^N labeled sample recorded at 310 K, pH 5.8 and 900 MHz. **b** Spectrum of a ^13^C/^15^N labeled sample (in complex with carbohydrate ligand) after 5 days measurements at 310 K, recorded at 310 K, pH 4.7 and 750 MHz. **c** Spectrum of a ^13^C/^15^N labeled sample after 6 weeks triple resonance measurements, recorded at 310 K, pH 5.8 and 600 MHz

**Fig. 3:**
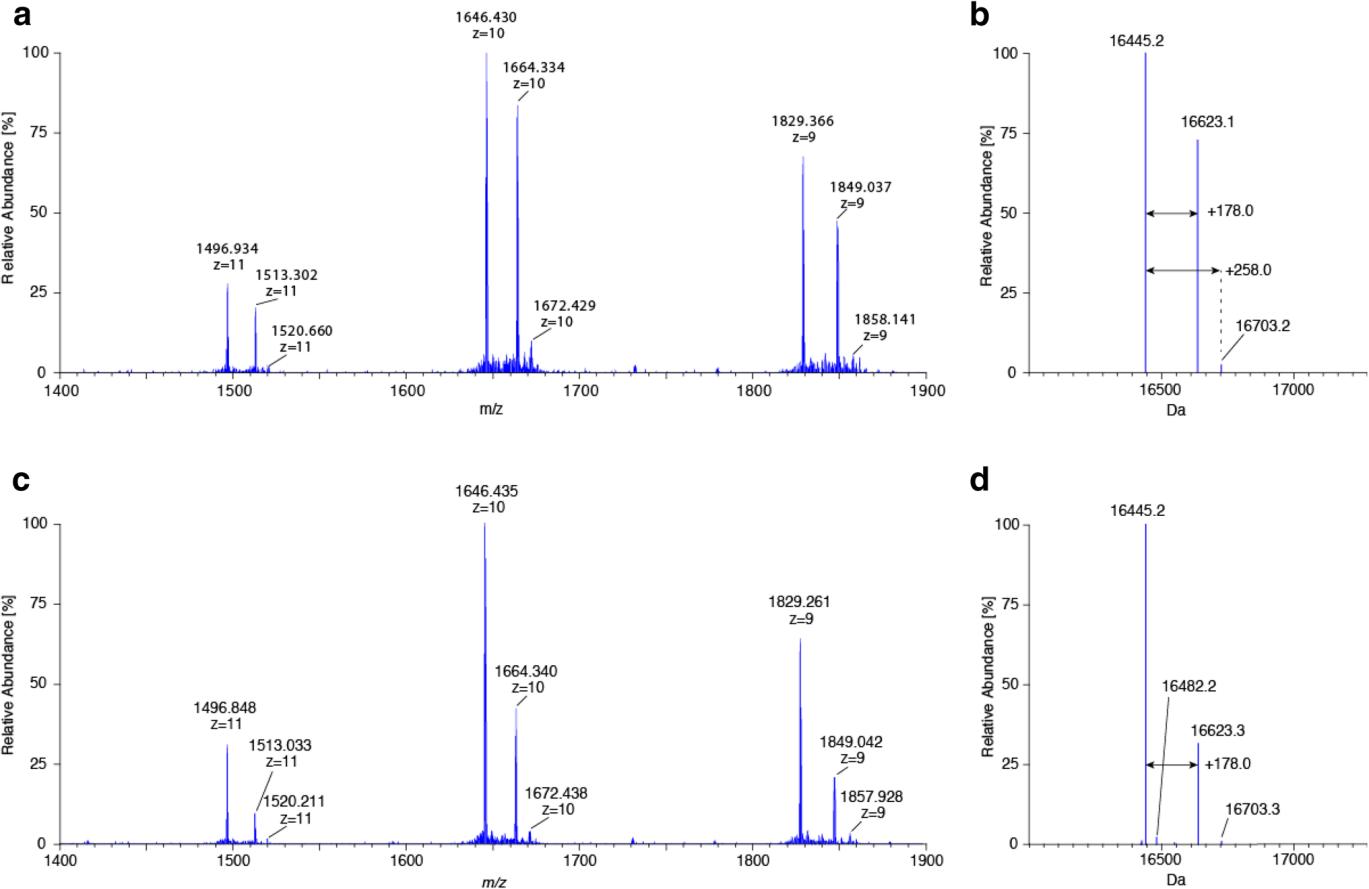
Mass spectra of the recombinant lectin CCL2 at natural abundance. **a** Zoomed raw mass spectrum of + 9 to + 11 charge states of recombinant CCL2 expressed in M9 minimal medium. **b** Deconvoluted mass spectrum of CCL2 expressed in M9 minimal medium. **c** Zoomed raw mass spectrum of + 9 to + 11 charge states of recombinant CCL2 expressed in LB medium. **d** Deconvoluted mass spectrum of CCL2 expressed in LB medium

In NMR spectra of recombinant CCL2 in a ^13^C/^15^N labeled form (Schubert et al. 2012) we observed quite intense and sharp ^1^H–^13^C chemical shift correlations with ^13^C chemical shifts between 70 and 78 ppm (Fig. 2), and since it was not possible to get rid of those signals by changing the purification scheme, we considered that these signals arose from a post-translational modification. Mass spectra of unlabeled CCL2, expressed either in M9 minimal medium or LB medium, showed a signal at 16445.2 Da (Fig. 3) indicating the cleavage of Met1 and a signal corresponding to [M–Met1 + 178 Da], which is indicative for gluconoylation (Geoghegan et al. 1999). However, to unambiguously confirm the presence of the gluconoyl group, we undertook a detailed NMR analysis.

**Fig. 4:**
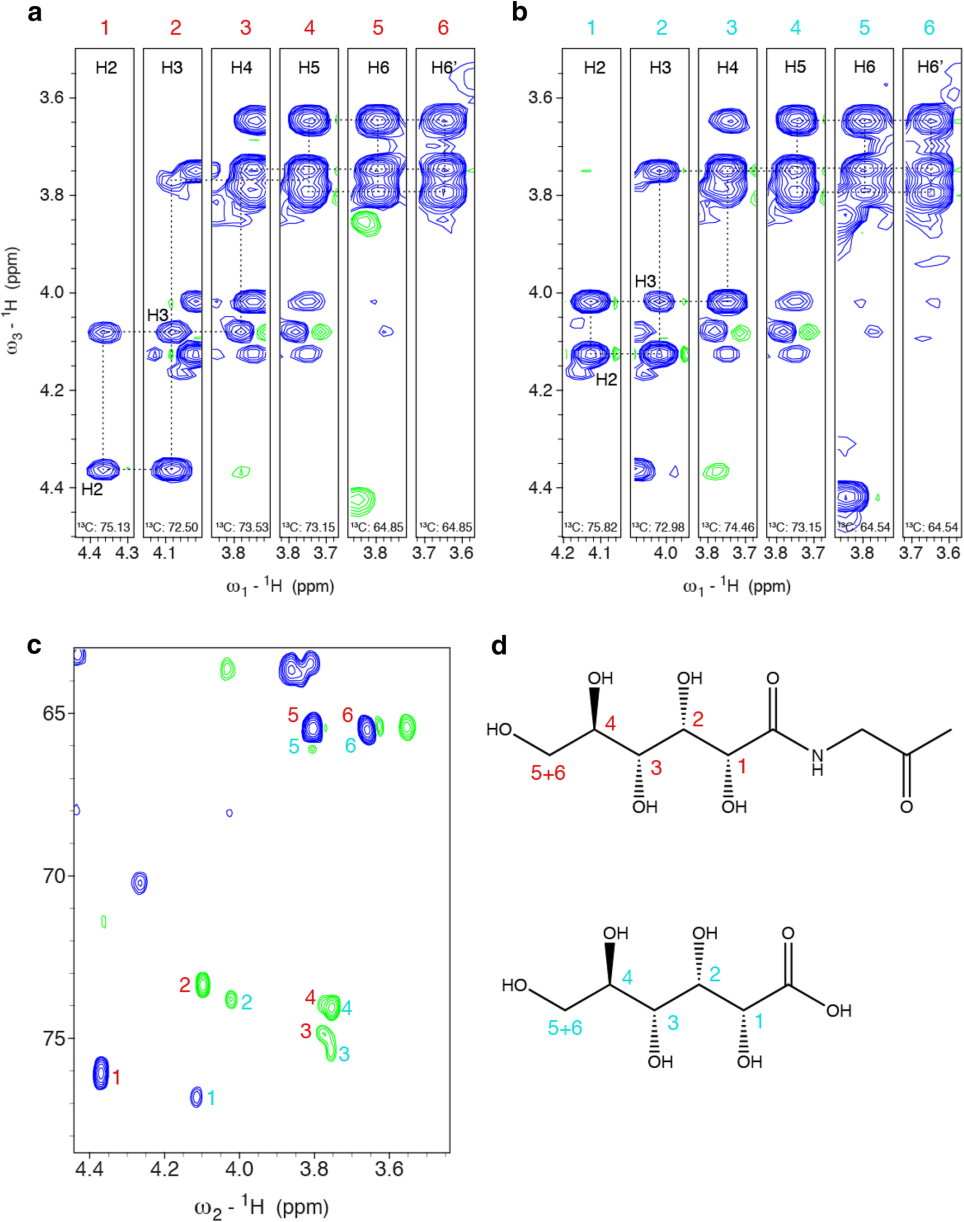
Three-dimensional HC(C)H-COSY of a CCL2 sample containing both the initial gluconoyl set of signals and those of the hydrolysis product. **a** Strips showing the correlations of the gluconoyl spin system. Beside the positive signals in blue, few negative truncation artifacts of signals in other planes are visible in green. **b** Strips of the newly appearing spin system. The strips 4–6 are identical for both spin systems due to overlapping frequencies. **c** Constant-time ^1^H–^13^C HSQC recorded with constant time period of 26.6 ms, resulting in opposite signs for correlations of CH_n_, depending on the number of ^13^C neighbors. A CH_n_ group with one ^13^C neighbor gives positive signals (blue), whereas one with two ^13^C neighbors gives negative signals (green). The labels at the signals correspond to the labels of the strips in the 3D HC(C)H-COSY. **d** Chemical structures of the identified N-terminal gluconoyl modification and its hydrolysis product gluconate. The labels of the observed signals are assigned to the corresponding atoms

^1^H–^13^C HSQC spectra of the recombinant lectin CCL2 (Schubert et al. 2012) showed the above mentioned sharp ^1^H–^13^C correlations (Fig. 2) in a region, where carbohydrate signals typically occur. The topology of the spin system could be studied by a 3D HC(C)H-COSY spectrum (Fig. 4a), whose analysis was, however, complicated by a second set of signals that appeared and turned out to be a hydrolysis product. Unfortunately, the two sets of signals partially overlapped (Fig. 2b). However, fresh samples contained only one set of signals (Fig. 2a), but two species were observed already after 2 days at 310 K (Fig. 2b). An aged sample that was used for several weeks of triple resonance experiments at 310 K showed only a single set of chemical shifts corresponding to the newly appeared signals. ^1^H–^13^C correlations in an edited ^1^H–^13^C HSQC revealed that the initial spin system consists of one CH_2_ and four CH groups (Fig. 2a). A constant-time ^1^H–^13^C HSQC, giving positive signals when an even number of ^13^C neighbors is present and negative signals in case of an odd number of ^13^C neighbors, revealed that most CH groups are part of a linear carbon chain (two ^13^C neighbors), one terminal CH_2_ and one terminal CH resulting in a spin system CH–CH–CH–CH–CH_2_ (Fig. 4c), in agreement with the topology of the gluconoyl carbon chain. Moreover, the obtained chemical shifts are comparable to those of gluconamide (Taravel and Pfannemuller 1990), *N*-octyl-gluconamide (Taravel and Pfannemuller 1990) and *N*-hexyl-gluconamide (Carter et al. 2000) (Table 1). They also seem to fit with the signals of a gluconoylated unlabeled peptide, whose HSQC was recently published, unfortunately without mentioning the chemical shift values (Martos-Maldonado et al. 2018). In agreement with the mass spectra that only show traces of [M–Met1 + 258 Da] (Fig. 3), we did not observe signals originating from 6-phosphogluconoyl moieties, indicating that the phosphate group was effectively cleaved after coupling of the 6-phosphogluconolactone to the protein N-terminus (Fig. 1).

**Table 1:** Comparison of the chemical shifts observed in this work (measured at 310 K) and previously reported chemical shifts of gluconamides and gluconate (Taravel and Pfannemuller 1990; Carter et al. 2000; Pallagi et al. 2010)

Nucleus	Gluconoyl at 310 K, this work	Gluconamide at 303 K, D_2_O reported by Taravel and Pfannemuller^a^	*N*-octyl-d-gluconamide at 303 K, D_2_O reported by Taravel and Pfannemuller^a^	*N*-hexyl-d-Gluconamide 330 K, DMSO, by Carter et al.^b^	Gluconate at 310 K, this work	Gluconate at 303 K reported by Taravel and Pfannemuller^a^	Gluconate at 298 K, pH 6 reported by Pallagi et al.^c^
C1	178.0	180.4	176.8	174.6	n.d.	181.4	181.5
C2	76.0	76.0	76.3	76.0	76.9	76.9	76.9
H2	4.37			4.23	4.11		3.95
C3	73.3	73.1	73.2	72.4	73.9	73.8	73.8
H3	4.10			4.15	4.02		4.03
C4	74.6	75.0	75.1	74.8	75.5	75.4	75.4
H4	3.79			3.75	3.76		3.78
C5	73.9	73.9	74.0	73.8	74.1	74.0	74.1
H5	3.75			3.75	3.76		3.74
C6	65.4	65.4	65.5	65.7	65.6	65.4	65.5
H6	3.81			3.83	3.81		3.82
H6′	3.66			3.64	3.66		3.65
N (N-term. glycine)	109.9		–	–	–	–	–
HN	8.44		–	–	–	–	–

^a 13^C referencing was different, the values in this table correspond to the reported values + 3.0 ppm

^b^ Personal communication by Dr. Donald Kiely, ^13^C referencing was different, the values in this work correspond to the reported values + 2.2 ppm; also ^1^H referencing was different, the values in this table correspond to the reported values + 0.25 ppm

^c 13^C referencing was different, the values in this table correspond to the reported values + 3.1 ppm. Also ^1^H referencing was different, the values in this work correspond to the reported values + 0.25 ppm

**Fig. 5:**
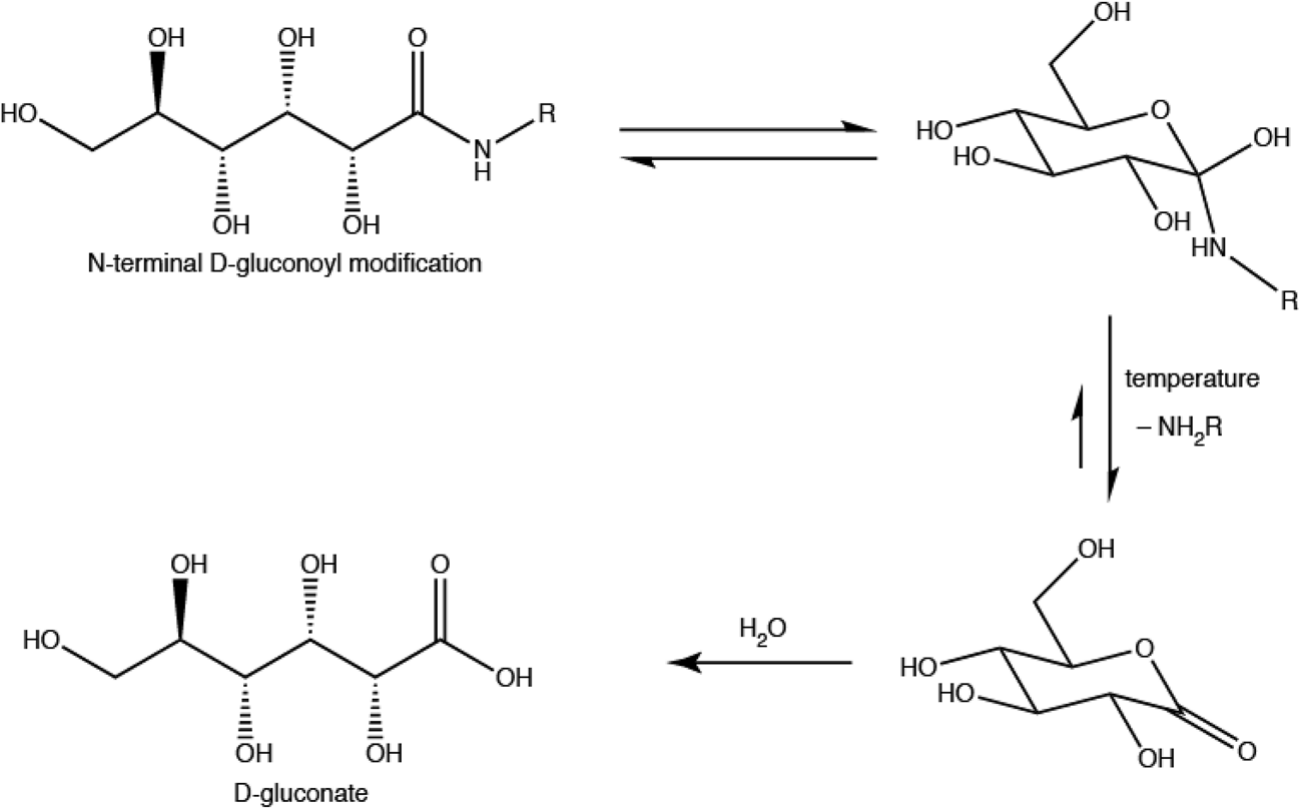
Suggested reaction steps leading to the hydrolysis of gluconoylated protein chains

The second set of chemical shifts that appear over time was analyzed using 2D ^13^C–^1^H correlations, 2D TOCSY and 3D HCCH-TOCSY spectra yielding also a spin system CH–CH–CH–CH–CH_2_. Inspired by the description of the hydrolysis of gluconamide to gluconate at elevated temperatures (Taravel and Pfannemuller 1990), we realized that the newly appearing signals show identical ^13^C chemical shifts as gluconate (Table 1; Fig. 5). In addition, the observed ^1^H chemical shifts match to previously reported ones for gluconate (Pallagi et al. 2010).

### Site and level of gluconoylation

**Fig. 6:**
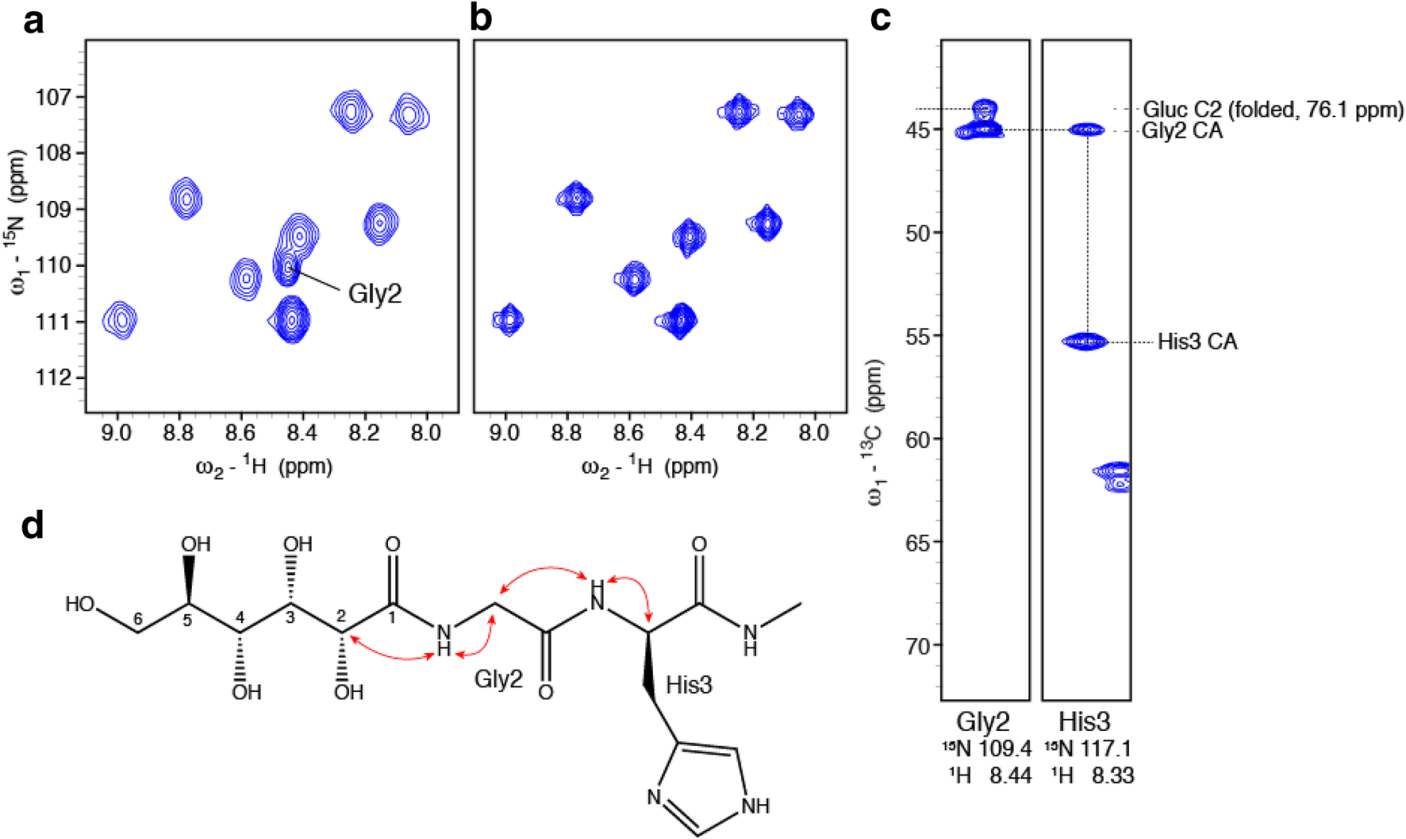
Nearly quantitative gluconoylation in fresh CCL2 samples. **a** ^15^N-HSQC of a fresh sample measured at 600 MHz and 310 K. The amide of Gly2 is indicated. **b** ^15^N-HSQC of a sample after several weeks of measurement at 310 K at 900 MHz. **c** Strips of a 3D HNCA spectrum of a freshly prepared CCL2 sample (in complex with carbohydrate ligand). **d** Schematic illustration of the correlations observed in the 3D HNCA

In a fresh sample of CCL2, a ^1^H–^15^N correlation of Gly2 is visible in a ^1^H–^15^N HSQC spectrum (Fig. 6a), which however, disappears over time (Fig. 6b). In a 3D HNCA spectrum this amide correlates with two ^13^C resonances at 45.0 ppm and 44.0 ppm (Fig. 6c), which correspond, to the Cα of Gly2 and to a folded signal of C2 of the gluconoyl moiety (Fig. 6d) with a chemical shift of 76.1 ppm, respectively. This provides strong evidence that Met1 is absent and that the gluconoyl group is linked to Gly2.

Further, the ^15^N–^1^H correlation of Gly2 could be quantified. In a fresh protein preparation (Fig. 6a), the integral of the Gly2 signal showed volumes comparable to other amides, indicating nearly quantitative gluconoylation. However, after 1 week at 310 K the signal was completely lost (Fig. 6b). After hydrolysis, gluconate and a free N-terminus are formed. The latter does not show any observable amide correlation because the NH_3_^+^ group exchanges rapidly with protons of water leading to the disappearance of the Gly2 signal.

The amount of gluconoylation was also analyzed by mass spectrometry of unlabeled CCL2 expressed in *E. coli* growing either on M9 minimal or LB medium (Fig. 3). The protein originating from M9 minimal medium contained a larger amount of gluconoylation (45%) than the one originating from LB medium (25%). In accordance to the MS data, the ^1^H–^13^C HSQC spectrum of a sample prepared from unlabeled LB medium with the natural abundance of ^13^C showed only a small amount of gluconoylation (Supplementary Fig. S3) compared to the one prepared with M9 minimal medium (Fig. 2), thus confirming the enhancement of gluconylation in M9 minimal media.

### Other examples of protein gluconoylation

In addition to CCL2 we analyzed two domains of hnRNP A1 (Barraud and Allain 2013), and the tandem zinc knuckles of pluripotency factor Lin28 (residues 124–186) (Loughlin et al. 2012), which were all recombinantly expressed in *E. coli* using ^13^C/^15^N labeled M9 minimal medium. ^1^H–^13^C HSQC spectra of the two RRM domains of hnRNP A1 and also the Zn-knuckles of Lin28 (Supplementary Fig. S1) display exactly the same ^1^H–^13^C correlations as described for CCL2, confirming the presence of the gluconoyl modification. Similarly, after few days of incubation at 303 K, the signals of the hydrolysis product gluconate appeared (Supplementary Fig. S1).

The gluconoyl chemical shifts of these examples (Supplementary Table S1) are almost identical to the ones observed for CCL2 (Table 1). To further verify if pH and temperature have an influence on the chemical shifts, we measured spectra of CCL2 at pH 7.5 and at the temperature of 298 K and 310 K (Supplementary Fig. S4). Highly similar chemical shifts were obtained (Supplementary Table S1).

## Discussion and conclusions

The degree of gluconoylation of certain recombinant proteins expressed in *E. coli* depends on the type of medium. Expression in M9 minimal medium resulted in higher abundance of gluconoyl compared to the expression in LB medium, which is particularly important for the production of isotopically labeled proteins for NMR spectroscopy. One explanation might be that the high glucose concentration in the M9 minimal medium leads also to high glucose-6-phopsphate and, subsequently, to high 6-phosphogluconolactone concentrations, which is the metabolite that is able to react with the free N-terminus of proteins. In addition, the cells are more stressed when growing in minimal medium.

Concerning the protein sequence preferences of gluconoylation, Geoghegan et al. reported that sequences starting with (M)GXXHHHH with XX being SS, SA, AS or AA, were all highly susceptible to gluconoylation (Geoghegan et al. 1999). The CCL2 construct started with (M)GHHHHHHHH, which was chosen to obtain high affinity for Ni–NTA. However this tag seems to be extremely susceptible to gluconoylation, because we obtained nearly quantitative modification with a ^15^N-labeled sample. Interestingly, a recent work aiming at the optimization of the N-terminal acylation of proteins identified GHHHHHH as the sequence with the highest reactivity towards gluconolactone and full conversion in vitro (Martos-Maldonado et al. 2018). The RRM domains of hnRNP A1 and also the Zn-knuckles of Lin28, which all started with (M)GSSHHHH showed significant gluconoylation, in agreement with Geoghegan et al. Gluconoylation is a non-enzymatic reaction that seems to be catalyzed by imidazoles in the vicinity following a general base catalysis (Martos-Maldonado et al. 2018). The more His residues that follow the N-terminus, the higher are the obtained gluconoyl yields. This might be explained by the fact that the pK_a_ of His is decreased in poly-His stretches making it a better base catalyst (Watly et al. 2014). Having a Gly with a highly mobile backbone before a stretch of histidines seems to enhance gluconoylation, however, it was reported earlier that peptides starting directly with histidines are also highly reactive to gluconic acid δ-lactone (Martos-Maldonado et al. 2018).

The gluconoyl modification is not very stable, it slowly hydrolyzes, resulting in a free N-terminus and gluconate. We observed hydrolysis at pH values 4.7–6.5 within few days and previous studies reported the hydrolysis of a gluconoylated peptide and a protein at pH 7.5 over a period of 7 days (Martos-Maldonado et al. 2018). The hydrolysis pathway shown in Fig. 5 is, in principle, also a reaction in equilibrium, as the formed gluconolactone could potentially react again with a free N-terminus to reintroduce the gluconoyl modification. However, the hydrolysis of the lactone to gluconate is not reversible. Moreover, we exclusively observed increasing amounts of gluconate, not of gluconolactone, in our NMR spectra with time.

The presented chemical shift assignments of the gluconoyl moiety, which were obtained for flexible N-termini of folded proteins under slightly acidic conditions, will serve as a reference to unambiguously identify gluconoylation as PTM in any recombinant or isolated protein. In case of large proteins and severe line broadening, working under denaturing conditions might be an option. Several PTMs have been detected under such conditions with ^1^H–^13^C correlations (Schubert et al. 2015; Grassi et al. 2017; Peng et al. 2018). The chemical shifts of the hydrolysis product gluconate might slightly vary, because they depend on the pH and also on the presence of Ca^2+^ that can be chelated (Pallagi et al. 2010), but the gluconoyl chemical shifts should be widely independent of the pH.

There are biotechnological activities that try to suppress gluconoylation. The widely used *E. coli* strain BL21(DE3) displays particularly high concentrations of δ-6-phospho- and γ-6-phosphogluconolactone (Meier et al. 2012) compared to other strains like the K-12 MG1655. However, BL21(DE3) is highly optimized for recombinant protein expression, it has less active proteases and contains the T7 polymerase gene, which is required by many protein expression vectors. All these features make this strain an indispensible tool for the production of recombinant proteins. A possible solution to suppress gluconoylation while using BL21(DE3) cells relies on the overexpression of a heterologous phosphogluconolactonase that reduces the amount of active lactone and thus gluconoylation (Aon et al. 2008).

## Electronic supplementary material

Below is the link to the electronic supplementary material.

Supplementary material 1 (PDF 482 KB)
