# Supplementary material for: The NMR signature of gluconoylation: a frequent N-terminal modification of isotope-labeled proteins
Source: J Biomol NMR. 2019 Feb 8;73(1):71–9. doi: 10.1007/s10858-019-00228-6 (PMC6441400; doi:10.1007/s10858-019-00228-6)
Supplement: Supplementary file 1 — Supplementary material 1 (PDF 482 KB) [file 10858_2019_228_MOESM1_ESM.pdf]

SUPPLEMENTARY MATERIAL

The NMR signature of gluconoylation - a frequent N-terminal modification of isotope-labeled proteins

David Schweida[1], Pierre Barraud[2,3], Christof Regl[1,4], Fionna E. Loughlin[2,5], Christian G. Huber[1,4], Chiara Cabrele[1,4], Mario Schubert[1,2,4*]

[1] Department of Biosciences, University of Salzburg, Billrothstr. 11, 5020 Salzburg, Austria

[2] Institute of Molecular Biology and Biophysics, ETH Zürich, CH-8093 Zürich, Switzerland

[3] Institut de biologie physico-chimique (IBPC), UMR 8261 CNRS, Université Paris Diderot, 13 rue Pierre et Marie Curie, 75005 Paris, France.

[4] Christian Doppler Laboratory for Innovative Tools for Biosimilar Characterization, University of Salzburg, Hellbrunnerstrasse 34, 5020 Salzburg, Austria

[5] Monash Biomedicine Discovery Institute, Department of Biochemistry & Molecular Biology, Monash University, Victoria 3800, Australia

Content:

Supplementary Figures S1 to S4	page 2
Supplementary Table S1	page 6
Supplementary References	page 7

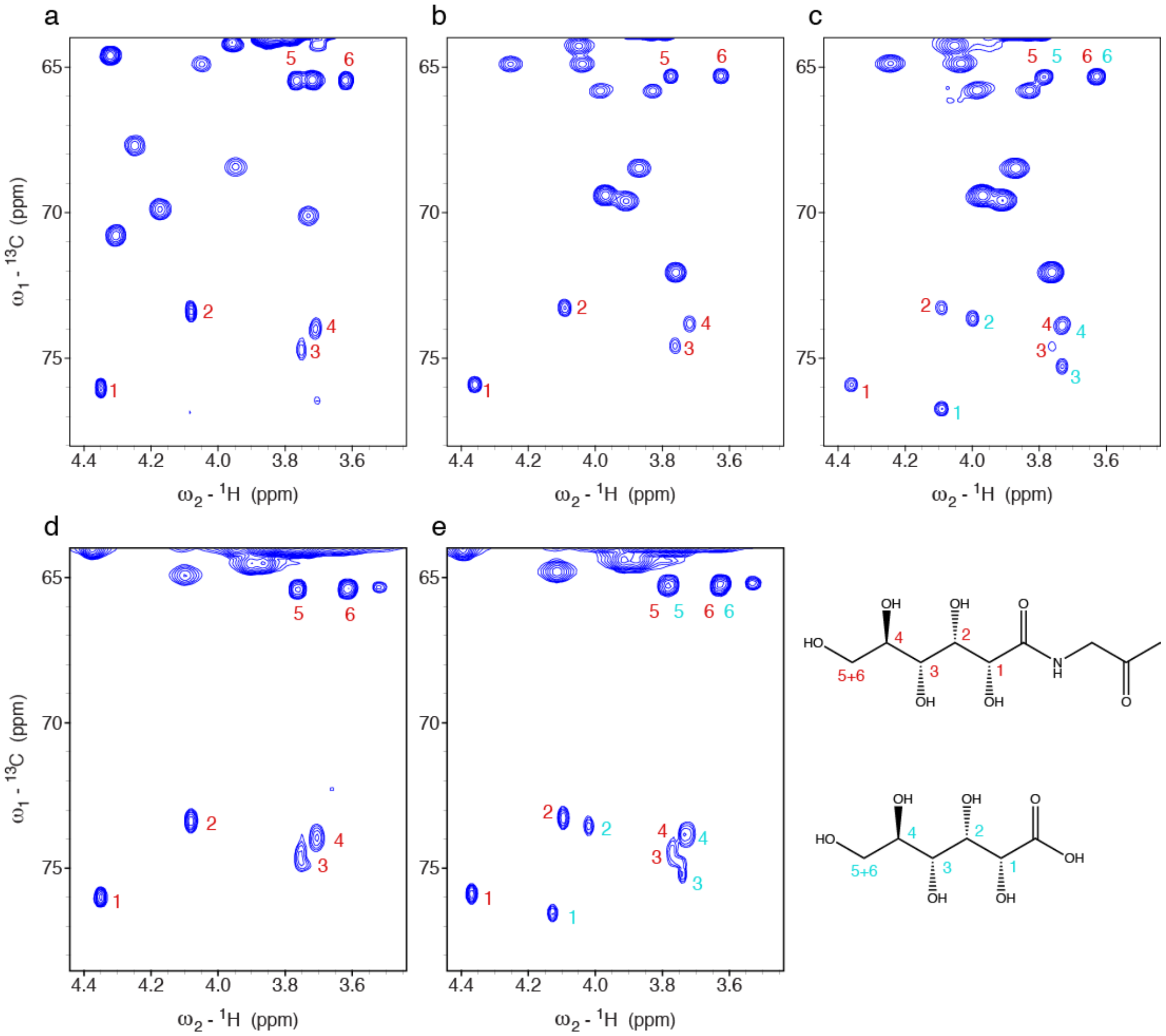

Supplementary Figure S1. ^{1}H-^{13}C HSQC spectra of a variety of additional ^{13}C/^{15}N labeled proteins. (a) Spectrum of a fresh sample of the RRM1 domain of hnRNP A1 in complex with the RNA UUAGGUC [1] recorded at 303 K, pH 6.5 and 750 MHz. (b) Spectrum of a fresh sample of the RRM2 domain of hnRNP A1 in complex with the RNA UCAGUU [1] recorded at 303 K, pH 6.5 and 900 MHz. (c) Spectrum of the same sample of panel b recorded after 5 days of triple resonance measurements at 303 K, pH 6.5 and 900 MHz. (d) Spectrum of a fresh sample of the two zinc knuckles of Lin28 in complex with the RNA AGGAGAU recorded at 303 K, pH 5.6 at 700 MHz in [2]. (e) Spectrum of the same sample of panel d after 4 days at 303 K.

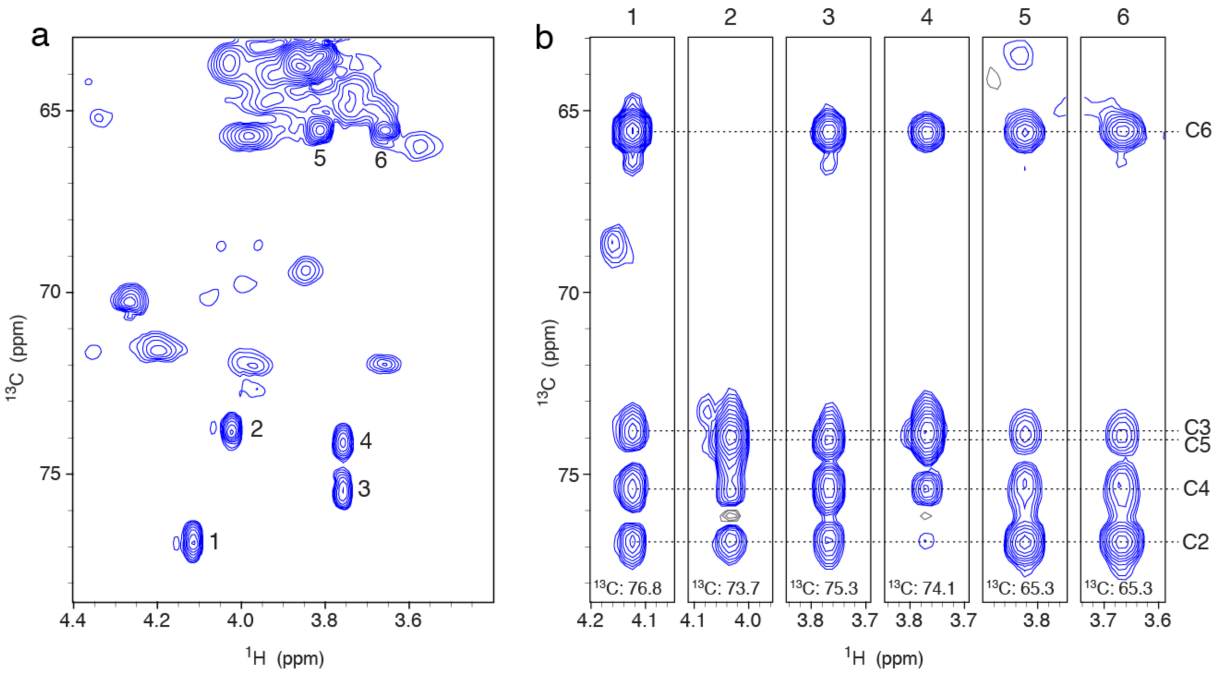

Supplementary Figure S2. Assignment of the hydrolysis product gluconate. (a) 2D ^{1}H-^{13}C HSQC of ^{13}C/^{15}N-labeled CCL2 (pH 5.8) expressed in M9 minimal medium after 6 weeks of NMR measurements at 310 K, recorded at 700 MHz and 310 K. (b) 3D (H)CCH-TOCSY spectrum of the same sample as in panel a.

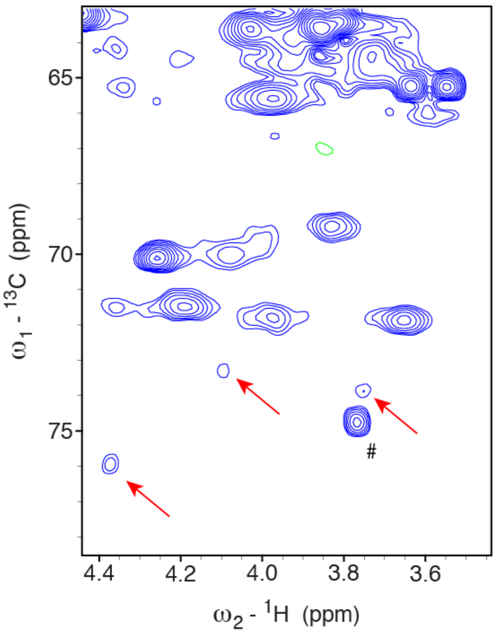

Supplementary Figure S3. ^{1}H-^{13}C HSQC of unlabeled CCL2 expressed in LB medium (pH 5.8), measured at ^{13}C natural abundance, at 600 MHz and 310 K. The arrows indicate characteristic and well isolated signals of gluconoyl that are present with low intensity.

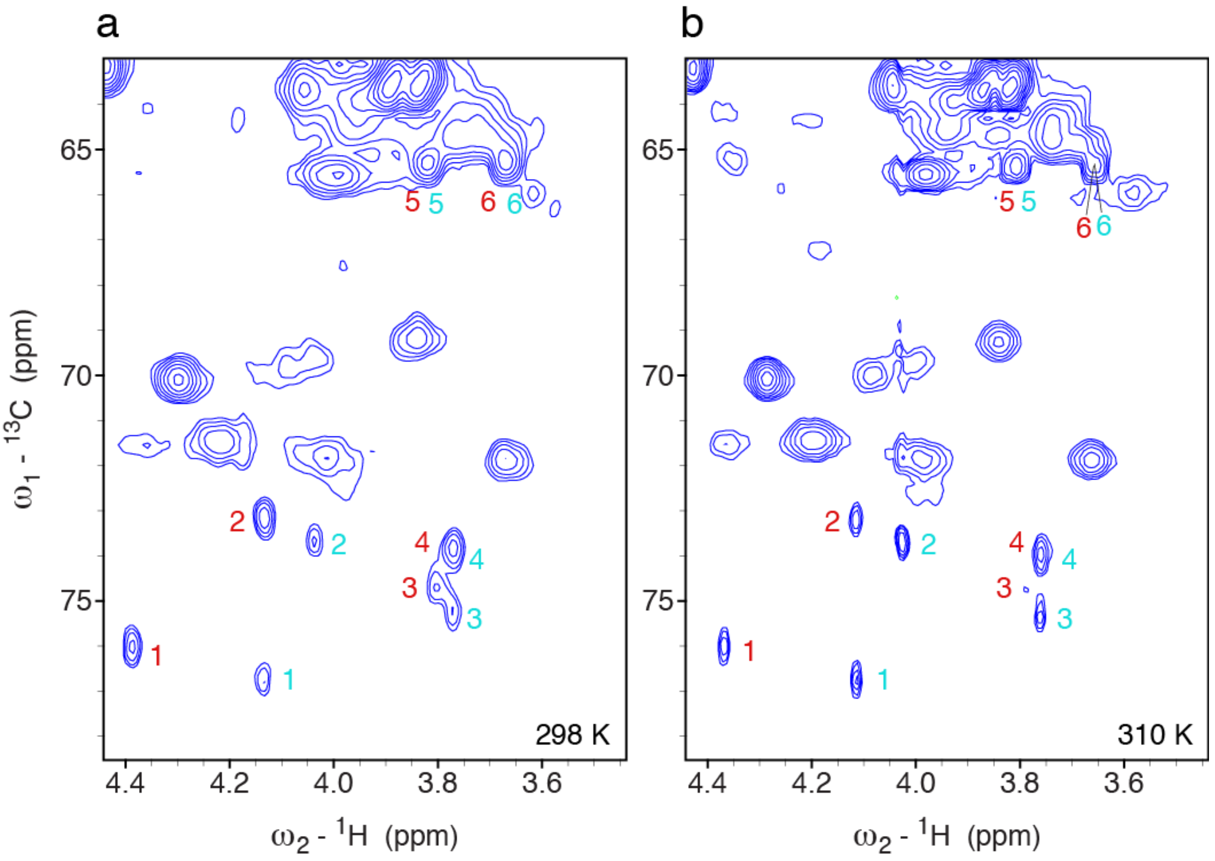

Supplementary Figure S4. ^1H-^{13}C HSQC of ^{13}C/^{15}N-labeled CCL2 (pH 7.5, 0.25 mM) measured at 600 MHz and two different temperatures. (a) Spectrum measured at 298 K with 4 transients and 1024×350 points. (b) Spectrum recorded at 310 K with 16 transients and 2024×512 points.

Supplementary Table S1. ^{1}H and ^{13}C chemical shifts of gluconoyl and gluconate at different pH values and different temperatures.

	Gluconoyl				Gluconate			
Nucleus	pH 5.6<br>303 K	pH 6.5<br>303 K	pH 7.5<br>310 K	pH 7.5<br>298 K	pH 5.6<br>303 K	pH 6.5<br>303 K	pH 7.5<br>310 K	pH 7.5<br>298 K
C2	75.9	75.9	76.0	76.0	76.7	76.7	76.8	76.8
C3	73.3	73.3	73.2	73.2	73.6	73.6	73.7	73.7
C4	74.5	74.6	74.7	74.7	75.2	75.3	75.4	75.2
C5	73.9	73.8	74.0	73.8	73.9	73.9	74.0	73.8
C6	65.3	65.3	65.4	65.3	65.3	65.3	65.4	65.3
H2	4.36	4.36	4.37	4.39	4.13	4.09	4.11	4.14
H3	4.09	4.09	4.12	4.13	4.02	4.00	4.03	4.04
H4	3.76	3.76	3.79	3.80	3.74	3.73	3.76	3.77
H5	3.72	3.72	3.76	3.77	3.73	3.73	3.76	3.77
H6	3.62	3.63	3.66	3.67	3.62	3.63	3.66	3.67
H6	3.77	3.78	3.81	3.82	3.77	3.79	3.81	3.82

SUPPLEMENTARY REFERENCES

1. Beusch, I., Barraud, P., Moursy, A., Clery, A. & Allain, F.H.T. Tandem hnRNP A1 RNA recognition motifs act in concert to repress the splicing of survival motor neuron exon 7. *Elife* **6**(2017).
2. Loughlin, F.E. et al. Structural basis of pre-let-7 miRNA recognition by the zinc knuckles of pluripotency factor Lin28. *Nature Structural & Molecular Biology* **19**, 84-U105 (2012).
